# Anti-Influenza Neuraminidase Inhibitor Oseltamivir Phosphate Induces Canine Mammary Cancer Cell Aggressiveness

**DOI:** 10.1371/journal.pone.0121590

**Published:** 2015-04-07

**Authors:** Joana T. de Oliveira, Ana L. Santos, Catarina Gomes, Rita Barros, Cláudia Ribeiro, Nuno Mendes, Augusto J. de Matos, M. Helena Vasconcelos, Maria José Oliveira, Celso A. Reis, Fátima Gärtner

**Affiliations:** 1 Instituto de Investigação e Inovação em Saúde, Universidade do Porto, Porto, Portugal; 2 Institute of Molecular Pathology and Immunology (IPATIMUP), University of Porto, Porto, Portugal; 3 Instituto de Ciências Biomédicas de Abel Salazar (ICBAS), University of Porto, Porto, Portugal; 4 Faculty of Veterinary Medicine, Lusophone University of Humanities and Technologies, Lisbon, Portugal; 5 Animal Science and Study Central (CECA), Food and Agrarian Sciences and Technologies Institute (ICETA), Porto, Portugal; 6 Department of Biological Sciences, Faculty of Pharmacy, University of Porto, Porto, Portugal; 7 Institute of Biomedical Engineering (INEB), University of Porto, Porto, Portugal; 8 Department of Pathology and Oncology, Faculty of Medicine of the University of Porto, Porto, Portugal; The University of Hong Kong, HONG KONG

## Abstract

Oseltamivir phosphate is a widely used anti-influenza sialidase inhibitor. Sialylation, governed by sialyltransferases and sialidases, is strongly implicated in the oncogenesis and progression of breast cancer. In this study we evaluated the biological behavior of canine mammary tumor cells upon oseltamivir phosphate treatment (a sialidase inhibitor) *in vitro* and *in vivo*. Our *in vitro* results showed that oseltamivir phosphate impairs sialidase activity leading to increased sialylation in CMA07 and CMT-U27 canine mammary cancer cells. Surprisingly, oseltamivir phosphate stimulated, CMT-U27 cell migration and invasion capacity *in vitro*, in a dose-dependent manner. CMT-U27 tumors xenograft of oseltamivir phosphate-treated nude mice showed increased sialylation, namely α2,6 terminal structures and SLe(x) expression. Remarkably, a trend towards increased lung metastases was observed in oseltamivir phosphate-treated nude mice. Taken together, our findings revealed that oseltamivir impairs canine mammary cancer cell sialidase activity, altering the sialylation pattern of canine mammary tumors, and leading, surprisingly, to *in vitro* and *in vivo* increased mammary tumor aggressiveness.

## Introduction

Cancer remains a great social and economic burden in the Western world. Indeed, despite all efforts to reduce such affliction, the number of patients has been increasing exponentially in the past few years. Breast cancer in particular is the most common cancer in women and the most frequent cause of cancer-related death, mostly due to the development of distant metastases [1].

The mechanisms involved in the establishment of cancer colonies in distant organs is far from being understood, as are the actual reasons which lead to metastasis-related death. Thus, identifying and investigating currently clinically used drugs which might have an impact on tumor progression is mandatory [2]. For instance, by interfering with numerous cell pathways which are common or similar between pathogens and hosts, drugs such as rapamycin and niclosamide (that were initially used as antifungal and antihelmintic drugs respectively) have turned out to be promising anticancer agents [3, 4].

Oseltamivir phosphate is an anti-influenza drug that has become widely used as prophylactic therapy since the time of the H1N1 pandemics [5]. It is administered as the prodrug oseltamivir phosphate, which is converted by carboxyl esterase enzymes into the active oseltamivir carboxylate. Oseltamivir phosphate is a sialic acid analogue which interacts with and blocks the active sites of sialidase enzymes of the influenza virus, it binds to the virus enzymes, blocking their ability to cleave sialic acid residues on the surface of the infected cell which results in an inability to release progeny virions [6].

Some suggestions have been previously made regarding potentially relevant pharmacological effects of this and other inhibitors of viral sialidases used in the clinics, in human endogenous sialidases [7]. While low nanomolar concentrations of oseltamivir carboxylate are sufficient to block activity of viral sialidases, this drug demonstrated almost no appreciable inhibition of human sialidases [7, 8]. Nevertheless, conflicting results were obtained when oseltamivir phosphate was tested in cancer cells using both *in vitro* and *in vivo* models [9, 10]. Indeed, some observations pointed out a possible inhibitory effect of oseltamivir phosphate on endogenous sialidases of rats and mice [11–14]. More recently, it was suggested that oseltamivir phosphate had the ability to reverse the epithelial to mesenchymal (EMT) transition process and increase drug sensitivity of chemoresistant human cancer cells [10].

Sialylated glycans are epidemiologically associated with worse prognosis in different types of cancer, including breast cancer [15]. Sialic acids are acidic monosaccharides usually found in the terminal position of carbohydrate chains present in glycoproteins and glycolipids [16]. Through complex interactions with selectins and siglecs among other molecules, sialic acids are physiologically present in different types of cell-cell interactions. Sialic acids increase the strength of charge density on the whole glycan chain, due to the presence of their carboxylic acid moiety, associating to changes in glycans’ adhesion properties [17]. Sialic acids are transferred from a donor substrate to a glycan structure present on a given glycoconjugate by sialyltransferases [18, 19]. On the other hand, their removal from glycan chains is catalyzed by sialidases. The activity of these enzymes is believed to affect the conformation of glycoproteins, and therefore contribute to either increased recognition or masking of biologically relevant sites in molecules and cells [20].

The increase of sialylated Lewis-type blood group antigens such as Sialyl Lewis X (SLe(x)) and small truncated glycans such as Sialyl Tn (STn) are amongst the most common glycan alterations in cancer cells [21–24]. Several functional assays in which sialylated glycans are shown to play a role in increased migration and invasion both *in vitro* and *in vivo* are found in the literature [19, 24]. On the other hand, tampering with sialylation *in vitro* has also been shown t sometimes reduce the invasive capacity of cancer cells [25]. As a result, modulation of sialylated glycans has been advocated as a putative target for neo-adjuvant therapy in cancer [26]. However, due to the wide range and complexity of sialyltransferases and all other glycosyltransferases involved in the sialylation process, there are well-recognized difficulties with turning sialic acids into clinically relevant therapeutic targets [27, 28]. However, even though there are more than 20 known different sialyltransferases, there are only four identified and characterized mammalian sialidases [20]. Interestingly, all four known mammalian sialidases are homologous to those of viruses containing, in some cases, residues identical to those found within the active enzymatic site [29].

There is thus a pressing need to study neoplastic contexts in which oseltamivir phosphate could competitively inhibit mammalian sialidases [30]. Different outcomes are likely to arise and may depend on the target of inhibition present and the array of affected glycoconjugates [20]. In this work we have studied the effects of sialidase inhibition as a modulator of sialylation-related mechanisms of invasion in mammary cell tumors using both *in vitro* and *in vivo* approaches.

## Material and Methods

### Cell lines and culture conditions

In order to assess possible differences of oseltamivir phosphate treatment in benign and malignant cell lines, two mammary cell lines were used in this study: one adenoma-derived cell line (CMA07—established at our laboratory from a spontaneously occurring canine complex adenoma excised from a 6 year-old female dog for curative purposes with owners consent [31]) and one carcinoma-derived cell line (CMT-U27 – highly metastatic canine carcinoma cell line, kindly provided by Professor Eva Héllmen, from Sweden [32]). The two cell lines were cultured at 37°C in a humidified 5% CO_2_ incubator (Thermo Scientific) and maintained in RPMI 1640 medium with Glutamax and 25 mM Hepes (Gibco Life Technologies), supplemented with 10% fetal bovine serum (FBS) (Gibco Life Technologies) and 1% Penicillin Streptomycin (P/S)(Gibco Life Technologies).

### Sialidase activity assay

Higher sialylation levels are expected in malignant cells when compared with benign counterparts. This is as likely to be dependent on the activity both sialyltranferases and on sialidase [17]. To evaluate the effect of oseltamivir phosphate on the activity of sialidases, an *in vitro* assay using a modified sialic acid (4-methyl-umbelliferyl-Nacetylneuraminic acid-4-MuNana), was performed using CMA07 and CMT-U27 canine mammary tumor cells. Sialidase activity was determined by obtaining the metabolic conversion of the sialic acid analog, 4-MuNana, into the fluorescent compound methyl-umbelliferone (blue color), upon treatment with different doses of oseltamivir phosphate. Cells were grown in 12 mm circular glass until confluence and then incubated for 24 h with medium containing different oseltamivir phosphate concentrations (0.305 μM, 3.05 μM, 30.5 μM and 305 μM oseltamivir phosphate dissolved in PBS), and the vehicle of the drug (PBS) was used as control. After 24 hours of treatment, each 12 mm circular glass was placed on a slide and incubated in a 2μM 4-MuNana (2'-(4-Methylumbelliferyl)-α-D-N-acetylneuraminic acid) (Sigma, Saint Louis,USA) solution. Slides were immediately observed with epi-fluorescent microscopy under UV light (excitation wave length at 360 nm, and emission wave length at 440 nm), as has been previously described [33]. Slides were analyzed and images were taken with a Carl Zeiss fluorescent microscope (Carl Zeiss Microscopy).

### Cell morphology analysis

CMA07 and CMT-U27 cells were plated at a density of 1x10^4^ cells per well in 6-well plates, in triplicate. Three different oseltamivir phosphate concentrations were studied: 0.305 μM, 3.05 μM and 30.5 μM, and PBS was used as control. Analysis of cell confluence and morphology was performed using a contrast inverted microscope over a period of 7 days. Photographs were taken at days 0 and 7 under 200x magnification.

### Cell proliferation assay

CMA07 and CMT-U27 cells were cultured in 24-well plates in triplicate for each condition: 0.305 μM, 3.05 μM, 30.5 μM and 305 μM oseltamivir phosphate and PBS was used as control. Cells were counted every day for 7 days in a Neubauer’s chamber in a 1:2 dilution of cells in 0.4% trypan blue and cell count was done using the volume conversion factor for 1 mm3, which is 1x10^4^. This assay was repeated 3 times and growth curves were traced.

### Cell growth assay

Cell growth was determined in CMA07 and CMT-U27 cell lines using a commercially available kit CellTiter 96 AQueous One Solution reagent (Promega Corporation), and performed according to manufacturer’s instructions. Briefly, cells were plated in 96-well plates in triplicate (Orange Scientific), at a density of 5x10^3^ cells per well. After cell attachment, oseltamivir phosphate was added at 0.305 μM, 3.05 μM, 30.5 μM and 305 μM final concentrations and PBS was used as control. Cellular metabolism was measured by adding MTS tetrazolium reagent and absorbance was recorded at 490nm. Measurements were performed at 0, 2, 4, 6, 8, 10, 12, 24 and 48 hours. An additional control measurement was performed at time-point 0h, in a culture well without cells. The experiments were performed twice.

### TUNEL assay

CMA07 and CMT-U27 cell lines were cultured in 6-well plates and then treated with different concentrations of oseltamivir phosphate (0.305 μM, 3.05 μM, 30.5 μM and 305 μM, and PBS was used as control). After 24 hours of treatment, culture medium and tripsinized cells were collected and centrifuged for 10 minutes at 2000 rpm. Cells were washed in PBS and fixed in cold methanol for 20 minutes. After fixation, cells were ressuspended in 1 mL of PBS for cytospin procedure. Briefly, 100 μL of cell suspension were centrifuged in a cytospin3 centrifuge using polilysine coated slides. Slides were then used for *in situ* cell death detection using a commercially available kit (*In situ* cell death detection kit, fluorescein from Roche) based on labeling DNA double strand breaks (TUNEL technology), according to manufacturer's instructions. Slides were observed under a fluorescence microscope using a 488nm excitation wavelength and percentage of dead cells was calculated by recording positive TUNEL cells in relation to total cells using the ImageJ software. This assay was performed twice.

### Wound-healing

The wound-healing assay was performed using a benign (CMA07) and a highly metastatic (CMT-U27) canine mammary tumor cell line in a time-lapse microscope. Briefly, 20x10^4^ cells were plated onto a 24-well culture plate and after reaching high confluence an artificial "wound" was made with a pipette tip. Culture medium was replaced with the different oseltamivir phosphate concentrations: 0.305 μM, 3.05 μM and 30.5 μM oseltamivir phosphate and PBS as control. Wound image acquisition was done with 5 minutes intervals for 48 hours, using the program Axio Vision Release 4.8.2. and converted in video. Treatment of cells with 305 μM oseltamivir phosphate was not performed due to its previously shown cytotoxicity. This assay was performed twice.

### Matrigel Invasion Assay

A matrigel invasion assay was performed to evaluate the invasive capacity of CMT-U27 cells, a good model to study cell invasion [34]. The matrigel inserts were re-hydrated with RPMI 1640 for 1 hour at 37°C in a humidified 5% CO2 incubator (Thermo Scientific). The media conditions were the same in the top and the bottom wells (RPMI 1640 medium supplemented with 10% of FBS and 1% of penicilin and streptimicin).

After insert rehydration, 1x10^5^ cells were seeded on Matrigel-coated chambers in the presence of different oseltamivir phosphate concentrations (0.305 μM, 3.05 μM, 30.5 μM oseltamivir phosphate) and PBS as control, and maintained in culture for further 6 hours (previously optimized time point for invasion assays using this cell line). The drug was used in the top wells. Following incubation, the content of each insert was removed and washed twice with PBS to remove non-invading cells. Invasive cells were fixed with cold methanol for 20 minutes, inserts were transferred onto slides (Industrial Quality) and mounted with Vectashield mounting medium with DAPI (Vector Laboratories). Invading cells were recorded by counting the number of cells present in the insert. These experiments were performed 3 times.

### Fluorescent cytochemistry

Cells were cultured in glass coverslips and the culture medium was supplemented with 0.305 μM, 3.05 μM and 30.5 μM oseltamivir phosphate and PBS as control, for 24 hours. Cells were then washed with PBS and fixed with cold methanol for 20 minutes. Following fixation, cells were re-hydrated with PBS and blocked with 10% BSA for 20 minutes. Plant lectins SNA (Biotinylated Elderberry bark lectin, B-1305, Vector Laboratories), MAL I (Biotinylated Maackia amurensis lectin I, B-1315, Vector Laboratories), and MAL II (Biotinylated Maackia amurensis lectin II, B-1265, Vector Laboratories) were diluted 1:300 in 5% BSA in PBS and incubated on slides for 1 hour at room temperature. Slides were then washed three times with PBS and incubated 1 hour with streptavidin-FITC. After two washes with PBS, slides were incubated for 10 minutes with DAPI (Sigma-Aldrich) in PBS and slides were mounted in Vectashield mounting medium (Vector Laboratories) for fluorescence analysis. Slides were analysed and images were taken in a Carl Zeiss fluorescent microscope (Carl Zeiss Microscopy).

### Western Blot analysis

Cells from CMA07 and CMT-U27 cell lines were grown to confluence in 6 well-plates and different concentrations of oseltamivir phosphate were added to the medium (0.305 μM, 3.05 μM and 30.5 μM oseltamivir phosphate). After 24 hours of incubation, cells were washed three times with PBS and lysed using RIPA lysis buffer (50 mM Tris HCl, pH 8; 150 mM NaCl; 1% NP-40; 0,5% sodium desoxicolate; 0,1% SDS) containing complete protease inhibitor cocktail (Roche), 1mM PMSF (phenylmethyl sulfonyl fluoride), and 1mM Na_3_VO_4_ (sodium orthovanadate). Protein concentration was determined using the biocinchoninic acid method from Pierce BCA Protein Assay Kit (Pierce/ Thermo Scientific), according to the manufacturer’s instructions.

The total protein extracts were resolved in 10% SDS-PAGE, transferred to a nitrocellulose membrane (Amersham Biosciences/GE Healthcare Life Sciences) and incubated with different biotinylatedlectins: MAL I (B-1315, Vector Laboratories), MAL II (B-1265, Vector Laboratories) and SNA (B-1305, Vector Laboratories) diluted 1:500 in 5% BSA (Sigma-Aldrich) in PBS with 0.05% Tween-20 (Sigma-Aldrich, U.S.A.). After washing with PBS 0,05% Tween-20, membranes were incubated with avidin-biotin-peroxidase complex kit (Vectastain ABC kit Standard, Vector Laboratories) for 1 hour at room temperature. For SLe(x) and STn analysis, membranes were incubated with mouse monoclonal antibodies, KM93 (Millipore) diluted 1:500 and TKH2 (gently offered by Dr H. Clausen from Denmark), followed by incubation with an HRP-conjugated anti-mouse secondary antibody for 1 hour. Analysis was done by chemiluminescence using the ECL Western blotting detection reagent and films (both from GE Healthcare). Western blot for actin diluted 1:4000 (Santa Cruz Biotechnology) was used as loading control.

### 2D gel electrophoresis

Differences in individual proteins are difficult to visualize using standard Western Blot analysis. Trying to overcome this, we have performed a 2D gels analysis which better discriminates protein glycoforms in protein extracts. Protein samples from CMA07 and CMT-U27 cells treated with 3.05 μM of oseltamivor and PBS-treated control cells were precipitated (ProteoExtract, Calbiochem) and resuspended in rehydration buffer (7M Urea, 2M Thiourea, 4% (v/v) CHAPS and 0.0002% Bromophenol Blue) with 0.2% of ampholyte and quantified (2D Quant Kit from GE Healthcare). Passive rehydration of the strips was performed overnight with 200μg of total proteins using IPG strips of pH 3–10 NL (ReadyStrip; 0.5 x3 x70 mm, Bio-Rad, Hercules) at room temperature. Isoelectric focusing was performed on Protean IEF cell (Bio-Rad) with an initial voltage of 250 V for 15 min, and then by applying a voltage gradient up to 4000V with limiting current of 50 μA per strip and temperature set at 20°C. The first dimension was concluded at 14–20 kVh.

Following the isoelectric focusing proteins were reduced and alkylated by incubation with 2% DL-dithiothreitol (DTT) followed by 2.5% Iodoacetamide in an equilibration buffer (6M Urea, 2% SDS, 0.002% Bromophenol Blue, 75mM Tris pH 8.8, 29.3% Glycerol) for 10 min each under gentle agitation. The strips were then packed in a 1% low gelling (1% agarose in running buffer—25 mM Tris, 192 mM Glycine, and 0.1% (w/v) SDS, pH 8.3; Bio-Rad) on top of a 10% acrylamide gel (acrylamide/bisacrylamide 37.5:1, 2.6% from Bio-Rad). Second dimension electrophoresis was performed in a Mini-Protean tetra cell system (Bio-Rad) using 1xTris/Glycine/SDS buffer at constant voltage of 125 V. Western blot analysis using SNA lectin was performed as described in the previous section.

### Animal Studies

Animal experiments were carried out in accordance with the European Guidelines for the Care and Use of Laboratory Animals, Directive 2010/63/UE and the National Regulation published in 2013 (Decreto-Lei n.° 113/2013 de 7 de Agosto). N:NIH(S)II-nu/nu nude mice, were housed, breeded and maintained at the Ipatimup Animal House, in a pathogen-free environment under controlled conditions of light and humidity. The following Humane Endpoints were established: any signals of distress, suffering or pain, body weight loss greater than 20–25% of the body mass, anorexia and moribund state, related or not to the experimental procedure.

### Experimental mice groups and drug treatment

Female NIH(S)II-nu/nu nude mice, aged 4–6 weeks, were orthotopically inoculated with 1 x 106 viable CMT-U27 canine breast cancer cells in the mammary fat pad using a 25 gauge needle. A total of 8 mice were inoculated. When nodules reached a volume of approximately 500mm3, mice (n = 8) were randomized and divided into control group (n = 4) and treatment group (n = 4).The animals received intraperitoneally (IP) dailly either 100 μL of PBS (control group) or 100mg/Kg of Oseltamivir phosphate (Tamiflu Roche) purchased from the pharmacy, diluted in PBS (treatment group) until time of death. Tumor size was measured using calipers, and tumor volume (mm3) was estimated by width x length x height. To observe metastization, primary tumors of all mice were surgically removed when a mean volume of ~1000–1500mm3 was reached. Mice were anesthetized by IP administration of 100 μL of a mixture containing 50 mg/kg of Ketamin (IMALGENE 1000) and 1 mg/kg of medetomidine hydrochloride (Medetor) and the tumor was excised. We used 2.5 mg/kg of atipamezole (Revertor) per mice to antagonize the effect of anesthesia. Mice were treated with an oral solution of 10 mg/kg of tramadol chloridrate (Tramal) every 8h for 24–48h to prevent pain. Animals were followed up after surgical excision of primary tumors for invasion and/or metastization signs.

All mice were euthanized according to the established Humane Endpoints and necropsied. Tissues were fixed in 10% buffered formalin, processed and embedded in paraffin. Three micrometer sections were cut and stained with hematoxilin & eosin (HE) for further histopathological and immunohistochemical analysis. Histopathological evaluation of primary tumors and respective metastases as well as inflammation assessment (counting the number of inflammatory infiltrates present in the whole xenograft) was performed independently by two pathologists.

### Histochemistry with lectins and immunohistochemistry

The expression of sialylated structures in CMT-U27 xenografts of mice treated with oseltamivir and controls were performed using plant lectins SNA, MAL I and MAL II.

Slides were deparaffinized and rehydrated followed by antigen recovery with boiling 0,005% Extran for 8 minutes. Endogenous peroxidase activity was blocked with 10% H2O2 in methanol solution for 10 minutes, and slides were blocked with 10% BSA in PBS (pH 7.4) for 30 minutes.

Then, tumor sections were incubated for 2 hours at room temperature with the biotinylated SNA, MAL I and MAL II (Vector Laboratories.), diluted at 1:250, 1:250 and 1:150 respectively in 5% BSA in PBS. Slides were then washed in PBS, incubated with the avidin-biotin-peroxidase complex (ABC) for 30 minutes and developed with diaminobenzidine tetrahydroxychloride substrate, DAB (SIGMA FAST). For immunohistochemistry, sections were incubated overnight at room temperature with the primary antibodies anti-Ki67 monoclonal antibody (MIB1 clone, Dako), anti-caspase-3 (5A1E clone, Cell Signalling), and anti-SLe(x) (KM93, Millipore) at 1:50, 1:100 and 1:200 respectively in 5% BSA in PBS. Slides were then washed in PBS, incubated with Novolink Max Polymer Detection System (1250 testes), Novocastra, (Newcastle, UK) for 30 minutes at room temperature and developed applying DAB of the same kit for 5 minutes.

The slides were then counterstained with hematoxylin, dehydrated, and mounted with Histomount (National Diagnostics). Negative controls, without lectins or antibodies, were included. All stained sections were examined with light microscopy and reviewed by three observers (de Oliveira J.; Ribeiro, C.; and Gartner, F.).

### Statistical analysis

Whenever appropriate, the results are presented as mean ± standard deviation. Statistical analysis was performed using One Way ANOVA (Analysis of variance) test. For MTS, cell proliferation, and cell growth assays multiple comparisons Dunnett method were used. Student t test was performed to evaluate the wound healing assay results. Multiple comparisons Tukey’s test was used to study cell matrigel invasion assay and multiple comparisons Bonferroni test was used for Tunel assay analysis. GraphPad Prism 5.02 version was used to perform these statistical analysis.

Imunohistochemical images of Ki-67 and caspase-3 expression were analyzed using the ImmunoRatio online analysis tools, under the high-power magnification in each area [35]. Tumor cases were divided into 3 categories according to the Ki67 score: less than 10% Ki67 positive cells (low), 10 to 50% Ki67-positive cells (moderate) and more than 50% Ki67-positive cells (high). To evaluate caspase 3 and SLe^x^ expression, tumors were grouped according to the percentage of immunoreactive cells throughout the whole lesion, into 3 classes: 0% (Negative); less than 5% (rare cells) or 6–10% (low). Regarding SNA and MAA I expression, a semi-quantitative analysis in which samples were scored according to the percentage of positive cells. Tumors were grouped into: less than 25% (low); 25 to 50% (moderate) and more than 50% (high). Then association hypotheses were tested, using Chi-square test for discrete variables. SPSS software (version 22.0) was used for statistical analysis.

## Results

### Influence of oseltamivir phosphate uptake by CMA07 and CMT-U27 cells on the endogenous sialidase activity *in vitro*

**Fig 1:**
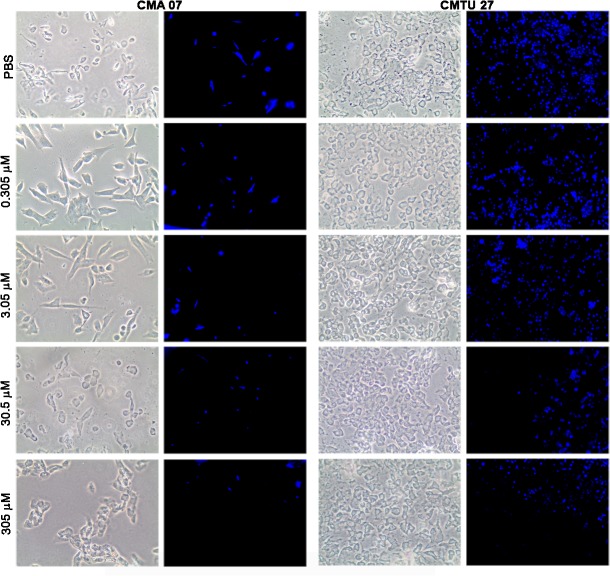
Sialidase activity assay upon oseltamivir treatment *in vitro*. CMA07 and CMT-U27 cells were treated for 24 hours with different concentrations of oseltamivir phosphate (0.305 μM, 3.05 μM, 30.5 μM and 305 μM) or PBS as control, and incubated with a 2 μM 4-MuNana solution. Slides were immediately observed under an epi-fluorescent microscope with UV light. Both cell lines showed decreased sialidase activity as assessed by the fluorescent methyl-umbelliferone, which results from metabolic conversion of the 4-MuNana.

Oseltamivir phosphate treatment impaired sialidase activity (neuraminic acid hydrolysis), as shown by a decrease of methyl-umbelliferone fluorescence in both cell lines (Fig 1). In addition, a decrease of sialidase activity was increasingly more evident in higher oseltamivir phosphate concentrations.

### Effect of oseltamivir phosphate treatment in CMA07 and CMT-U27 cells morphology, viability and programmed cell death

To evaluate the effect of different doses of oseltamivir phosphate on cell morphology, viability and programmed cell death, the *in vitro* assays below were performed.

Slight alterations in cell morphology were observed in CMA07 cells treated with oseltamivir phosphate. No major alterations in CMT-U27 cell morphology were observed (S1 Fig).

**Fig 2:**
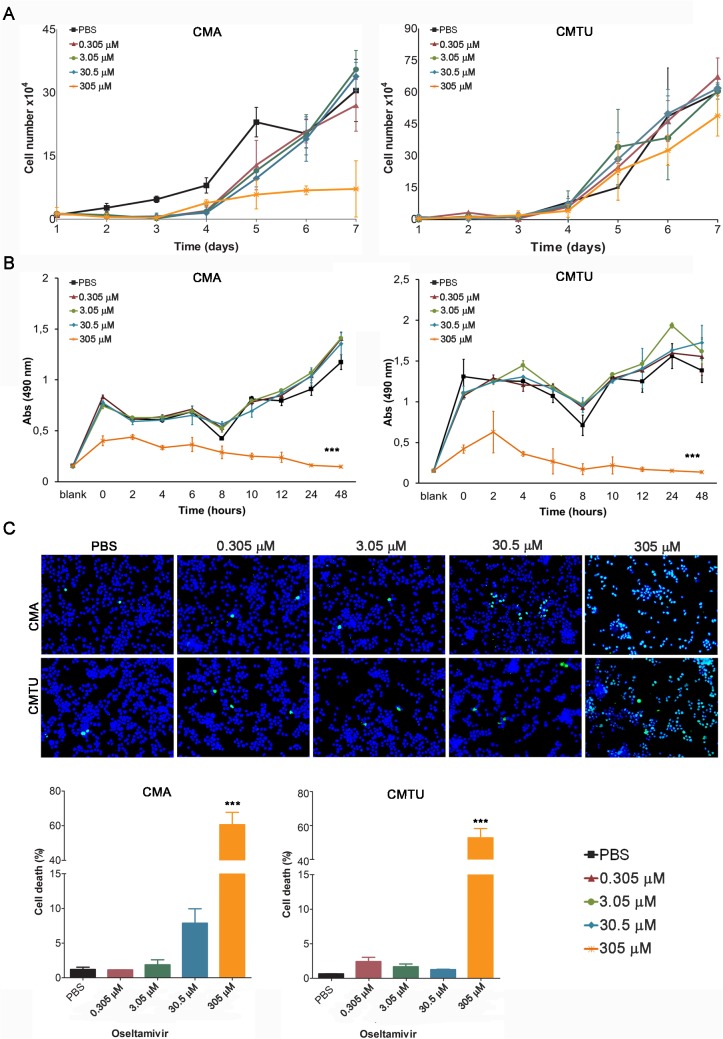
Cell growth, cell viability and programmed cell death following oseltamivir treatment. Cell growth assessed with Trypan blue (A) or with the MTS assay (B) and programmed cell death (C) were evaluated in CMA07 and CMT-U27 cells following treatment with oseltamivir phosphate (0.305 μM, 3.05 μM, 30.5 μM and 305 μM) or PBS as control. (A) Cells were cultured in the presence of different oseltamivir phosphate concentrations, and cell growth was monitored for 7 days by counting the number of viable cells daily. No significant differences in growth rates of treated cells were verified when compared with non-treated cells, except for a decreased growth in CMA cells following 4 days of 305 μM oseltamivir phosphate treatment. (B) Cells were cultured in the presence of the different oseltamivir phosphate concentrations, and cell metabolic activity was further monitored using an MTS assay, in a time course manner for 48 hours. CMA07 and CMT-U27 cells treated with 305 μM oseltamivir phosphate showed a significant decrease in their metabolic activity (***p<0.001) but no changes were verified with the lower concentrations. (C) CMA07 and CMT-U27 were treated with different concentrations of oseltamivir for 24h. Cells treated with 305 μM oseltamivir phosphate also showed a statistically significant increase in programmed cell death (***p< 0.001) following 24 hours of oseltamivir phosphate treatment but no alterations were verified with the lower concentrations (graphs show quantification of TUNEL images as shown above). Color lines specified at the end of the picture intend to represent the stated different concentrations for all graphs presented in this picture.

Statistical analysis showed no significant differences in the growth rate of both CMA07 (*p* = 0.6209) and CMT-U27 (*p* = 0.9929) cell lines, upon treatment with oseltamivir phosphate (Fig 2A). To further evaluate the effect of oseltamivir treatment on CMA07 and CMT-U27 cell viability, the MTS colorimetric assay, that determines cellular metabolic activity, was performed, for 48 hours. The metabolic activity of CMA07 and CMT-U27 cell lines was significantly decreased with 305 μM oseltamivir phosphate treatment (*p* = 0.005 and p<0.0001 respectively) using One Way ANOVA testes (Analysis of variance). In contrast, no statistically significant alterations were observed with 0.305 μM (p = 0.9781), 3.05 μM (*p* = 0.7436) and 30.5 μM (*p* = 0.9623) of oseltamivir phosphate treatments when compared with control cells (Fig 2B). Finally, to assess the effect of oseltamivir phosphate on CMA07 and CMT-U27 programmed cell death, and given that 305 μM oseltamivir phosphate treatment impaired cell metabolic activity, a programmed cell death measurement was performed with the TUNEL assay. Twenty-four hour oseltamivir phosphate treatment, specifically at 305 μM, significantly increased CMA07 (*p* = 0.0010) and CMT-U27 (*p =* 0.0002) DNA fragmentation, suggesting promotion of programmed cell death, when compared with lower oseltamivir concentrations, or with PBS (Fig 2C upper and lower panels).

Together, these results indicate that treatment with 305 μM of oseltamivir phosphate causes cytotoxicity in both cell lines. For this reason, this highest oseltamivir concentration was excluded from the subsequent *in vitro* assays.

### Effect of oseltamivir treatment on CMT-U27 migration and invasion capacities

To evaluate the effect of oseltamivir treatment on the migration and invasion capacities of mammary tumor cells, wound healing and matrigel invasion assays were performed.

**Fig 3:**
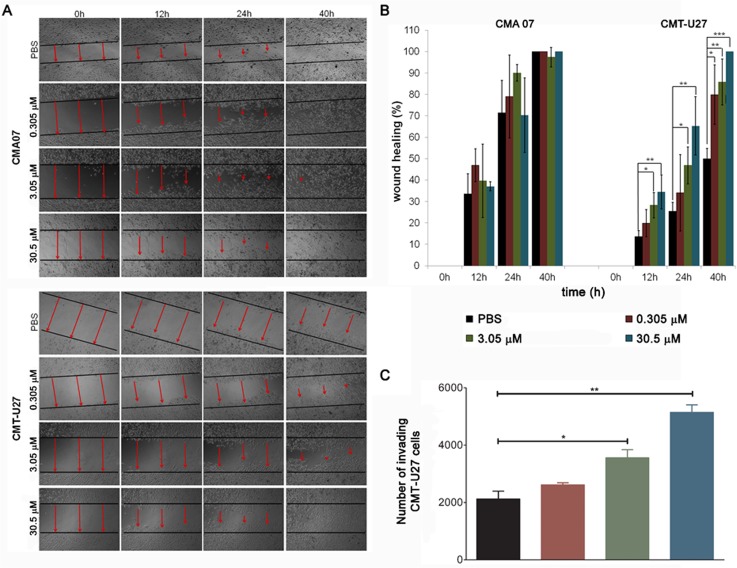
Motility and invasion capacity of canine mammary tumor cells treated with oseltamivir. CMA07 and CMT-U27 cell migration was evaluated by time-lapse following treatment with various oseltamivir phosphate concentrations (0.305 μM, 3.05 μM and 30.5 μM) or PBS as control. Representative images (A) and quantification of results (B) after wounding show an altered migration capacity upon treatment. CMT-U27 cells treated with 3.05 μM and 30.5 μM of oseltamivir phosphate for 12, 24 and 40 hours migrated faster than PBS-treated cells. Statistically significant differences were also observed for the lowest concentration tested (0.305 μM) at the longest time point tested (40 hours). Data are presented as means ±SEM from separated experiments. *p<0.05 **p<0.01; ***p<0.001. No significant differences were observed between oseltamivir phosphate-treated and control on CMA07 cells. (C) CMT-U27 invasive capacity *in vitro* was studied using a Biocoat Matrigel invasion chamber assay for 6 hours of oseltamivir phosphate treatment. Cells treated with 3.05 μM (*p<0.05) and 30.5 μM (**p<0.01) oseltamivir phosphate showed a significantly increased invasive capacity when compared to PBS-treated cells. For the 0.305 μM oseltamivir phosphate treatment no statistically significant differences were observed (ns *p*>0.05).

Oseltamivir phosphate treatment increased CMT-U27 cell migration, as evidenced by a decrease in the time needed to close the artificial wound compared to the control (Fig 3A). This increase in migration was dose-dependent and statistically significant (*p* = 0.032) for CMT-U27 cell line (Fig 3B) using One Way ANOVA (Analysis of variance). Our results thus indicate that oseltamivir phosphate increases CMT-U27 cells migrating ability. However no significant differences were observed in CMA-07 cell migraton after oseltamivir phosphate treatment.

Six-hour treatment with 3.05 μM (*p*<0.05) and 30.5 μM (*p*<0.01) oseltamivir phosphate significantly increased CMT-U27 *in vitro* invasive capacity, when compared with the PBS control (Fig 3C). Treatment with 0.305 μM oseltamivir phosphate (the lower concentration tested) also showed a non-significant (*p*>0.05) trend for a higher number of CMT-U27 cells invading the matrigel chamberThus, 6 hours of oseltamivir phosphate treatment promoted an increase in the invasive capacity of CMT-U27 cells in a dose-dependent manner (R^2^ = 0.836).

Altogether, these results suggest that oseltamivir phosphate decreased the activity of endogenous sialidases and enhanced CMT-U27 migration and *in vitro* invasive capacity. The observed increase in motility and invasive capacity might be related to a decrease in the activity of endogenous sialidases, possibly due to the role played by sialic acids in these processes.

### Effect of oseltamivir phosphate treatment in the expression of sialylated glycan structures by CMA07 and CMT-U27 cells

**Fig 4:**
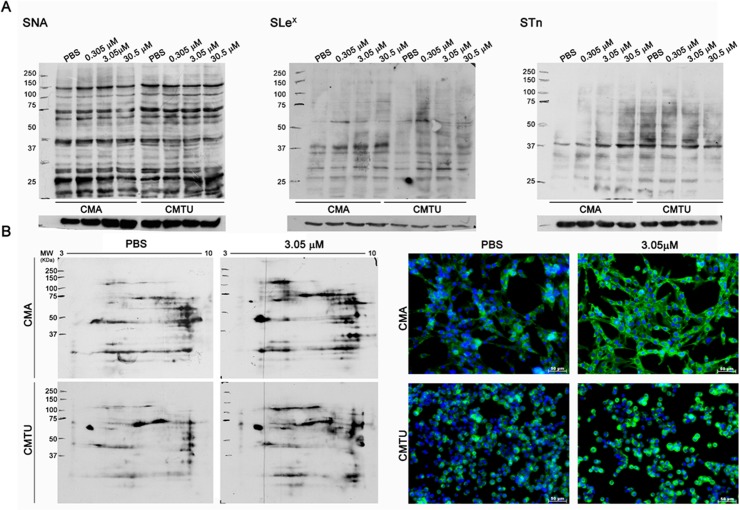
Analysis of sialylated glycan structures in canine mammary tumor cell lines treated with oseltamivir. (A) Expression of sialylated structures was evaluated by blot analysis using different lectins and monoclonal antibodies. Detection of α2,6 sialylated structures by SNA lectin blot showed increased expression of these structures in oseltamivir phosphate-treated CMA07 cells (0.305 μM, 3.05 μM and 30.5 μM), when compared control cells, mainly in the region of the 120 KDa molecular weight region. Analysis of SLe(x) and STn structures also demonstrated that they were increased in protein extracts from oseltamivir phosphate-treated cells. (B) 2D analysis of glycoproteins labeled with SNA better demonstrated an increased expression of α2,6 sialylated structures in 3.05 μM oseltamivir phosphate-treated cells when compared with PBS-treated cells. Finally, the broader expression of terminal α2,6 sialic acids structures was evaluated by SNA lectin fluorescent labelling upon oseltamivir phosphate treatment. Oseltamivir phosphate-treated CMT-U27 and CMA07 cells (3.05 μM) presented increased α2,6 sialic acids structures when compared with control cells (x20 magnification).

Western Blot analysis done to assess overall lectin binding, SLe(x) and STn expression in CMA07 and CMT-U27 protein extracts, showed few differences regarding MAL I—and MAL II – binding (recognizing α2,3 sialylated glycoconjugates) (S2 Fig). Oseltamivir phosphate treatment increased the expression α2,6-linked sialylated glycansin CMA07 and CMT-U27 cells (Fig 4A), within the 120 KDa molecular weight region. STn structures were increased upon oseltamivir treatment in CMA07 cel line but this was not verified in CMT-U27 cell line. In addition, oseltamivir phosphate treatment increased the expression of SLe(x) structures in different glycoproteins of CMA07 and CMT-U27 cells (Fig 4A). In 2D electrophoresis gels increased proteins sialylated with α2,6-linked structures were observed in the 120KDa region (Fig 4B). Fluorescence assays allowed us to have a broader view of differences in the cells glycome since lectins are able to bind not only to glycoproteins but also to glycolipids present in the cell.

Oseltamivir phosphate treatment induced a general increase in cells fluorescence of α2,6 sialylated glycoconjugates, recognized by SNA which was even more obvious than that observed in protein extracts alone. Together, these results suggest that oseltamivir phosphate treatment decreased the sialidase activity in CMA07 and CMT-U27 cells which in turn enhanced the expression of terminal sialic acids on several different glycoconjugates.

### Effects of oseltamivir treatment and expression of sialylated glycan structures in CMT-U27 xenografts

**Fig 5:**
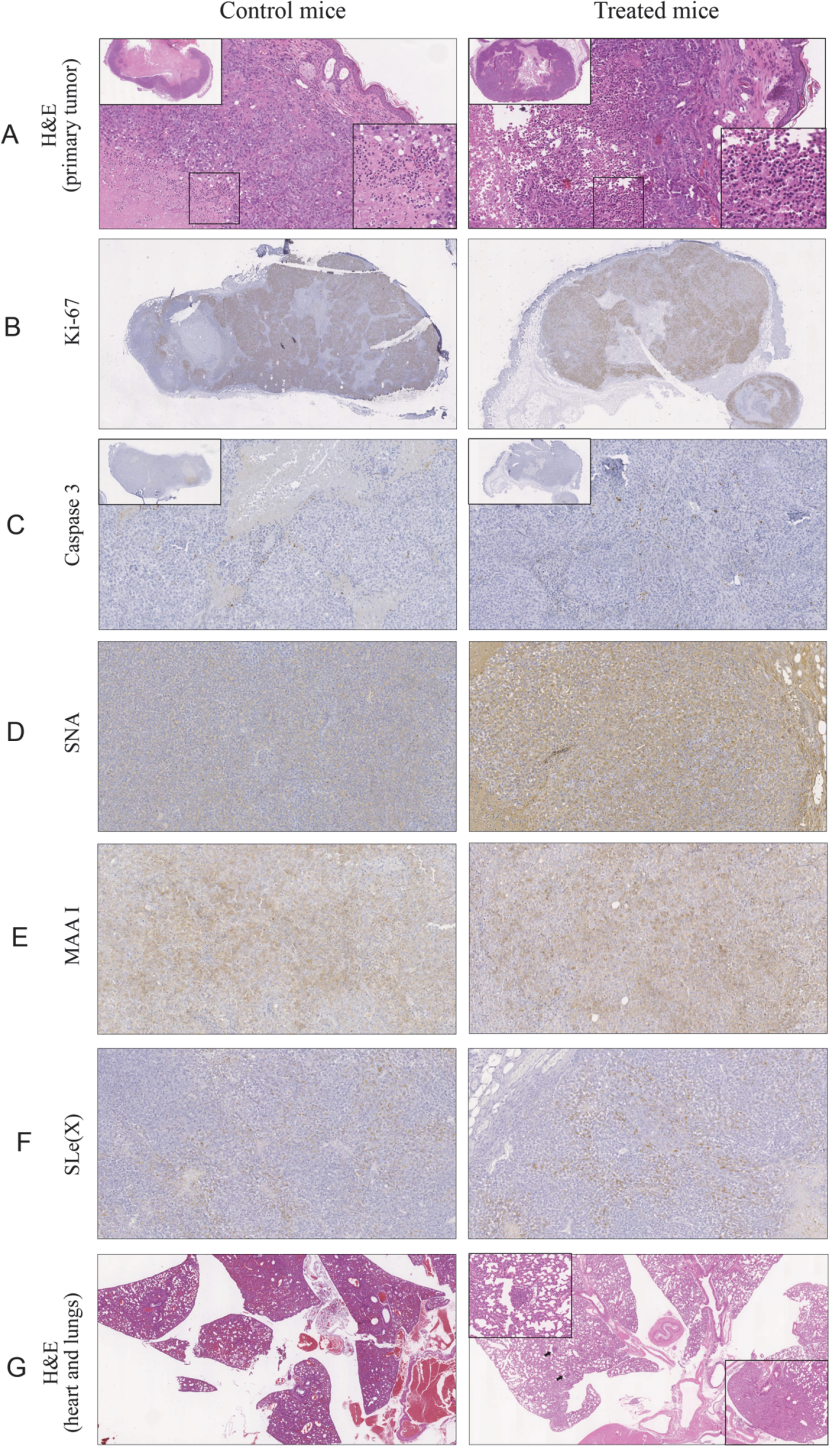
Study of the in vivo effect of oseltamivir treatment in CMT-U27 xenografts in female nude mice. CMT-U27 cells were xenografted into the mammary fat pad of 8 mice and divided in two groups according to treatment. Mice were treated daily (IP) with either 100mg/kg oseltamivir or PBS. Tumors were exised when reaching 1000–1500 mm^3^. (A) Oseltamivir-treated mice presented a more prominent inflammatory infiltrate when compared with non-treated animals. Representative images of Ki-67 (B) and caspase-3 (C) expression (brown colour). Histochemical staining with SNA (D) and MALI (E) lectins (brown colour) was used to study terminal α2,6 and Siaα2,3Galβ1-4GlcNAc sialic acid structures in CMT-U27 xenografts. Increased expression in α2,6 sialic acid terminal structures (*p* = 0.07) was observed in oseltamivir-treated when compared to non-treated mice. Overall siaα2-3Galβ1-4GlcNAc sialic acids structures (MALI) were less altered. (F) SLe(x) expression was also slightly increased in oseltamivir-treated when compared to non-treated mice. (G) Thorough analysis of lung tissues from both groups of mice showed a higher number of lung metastases in oseltamivir-treated when compared to non-treated animals (arrows).

Oseltamivir phosphate-treated mice presented significantly more inflammatory infiltrate in primary tumors (*p =* 0.01) (Fig 5A and Table 1). Ki-67 antigen and caspase-3 protein were used to assess CMT-U27 xenograft tumor cell proliferation and apoptosis respectively. Virtually no differences were found in Ki-67 and caspase 3 (*p* = 0.2) expression between oseltamivir-treated and non-treated mice (Fig 5B and 5C and Table 1). These observations are in agreement with the tendency observed regarding the tumors’ growth (S3 Fig), in which no significant differences were found between controls and treated animals.

**Table 1:** Effect of oseltamivir treatment in CMT-U27 xenografts in female nude mice.

	Untreated		Oseltamivir		*P value*
	N	%	N	%	
**Ki-67**					
High	4	100	4	100	^a^
**Caspase 3**					
Negative	1	25	0	0	
Rare cells	3	75	2	50	0.2
Low	0	0	2	50	
**Inflammation**					
Low	4	100	0	0	0.01
Moderate	0	0	4	100	
**Metastases**					
Absent	3	75	0	0	0.07
Present	1	25	4	100	
**MAL I**					
Low	1	25	0	0	
Moderate	2	50	2	50	0.5
High	1	25	2	50	
**SNA**					
Low	2	50	0	0	
Moderate	2	50	1	25	0.07
High	0	0	3	75	
**SLe** ^**x**^					
Negative	1	25	0	0	
Rare cells	2	50	0	0	0.09
Low	1	25	4	100	

^a^ – non applicable

The association hypothesis were tested using Chi-square test.

Then, in order to evaluate the effect of oseltamivir treatment in the glycosylation pattern of mammary tumor cells *in vivo*, terminal α2,6 and α2,3 sialic acid structures were assessed using SNA and MALI plant lectins in CMT-U27 tumor xenograft. Primary tumors of oseltamivir-treated mice showed increased expression in terminal α2,6 sialic acids structures (SNA) when compared with those of non-treated mice (Fig 5D and Table 1). This increase was however not statistically significant (*p* = 0.07) (Table 1). Siaα2,3Galβ1,4GlcNAc sialic acids structures (MALI) seemed more discretely increased in CMT-U27 xenografts of oseltamivir phosphate-treated when compared with non-treated mice, however no significant differences were found (*p* = 0.5) (Fig 5E and Table 1). A tremd for increased SLe(x) expression (*p* = 0.09) was also observed in primary tumors of oseltamivir phosphate-treated animals (Fig 5F). Of note, oseltamivir phosphate-treated mice bearing CMT-U27 xenografts presented earlier signs of disease (S4 Fig) and distress such as generalized weakness, cachexia and dyspnea. Two treated-animals presented ascitis. For ethical reasons the *in vivo* experience was ended and animals were humanely euthanized. A trend towards an increased number of lung metastases (*p* = 0.07) was observed in animals treated with oseltamivir phosphate (Fig 5G and Table 1).

## Discussion

Vast efforts are currently being made to better understand the mechanisms behind cancer spread and the risk factors involved [36]. Cancer cells have a profoundly different metabolism when compared with their normal cellular counterparts [37]. This raises concerns regarding overall exposure to carcinogens, namely the effects of commonly used drugs with increased cancer risk, such as people bearing preneoplastic lesions or even those bearing undiagnosed low-invading capacity cancers. Moreover, it is currently well-acknowledged that radiotherapy, chemotherapy, hormonal and monoclonal antibody-based anticancer therapies confer increased patient survival, however often eliciting activation of otherwise silenced intracellular pathways involved in drug resistance and cancer cell invasion [38]. Such secondary effects render the effects of commonly used drugs even more unpredictable on cancer patients undergoing remission. Nonetheless, these concerning issues remain unsolved since ethical constraints preclude clinical trials of marketed labelled drugs in cancer patients.

Domestic animals bearing mammary tumors are not only very interesting epidemiological models but also good models to monitor the natural course of the disease [39]. The lack of chemo- and radiotherapy protocols with proven clinical evidence makes surgery the almost single utilized approach [40]. Thus, this should be considered as a good spontaneous pre-clinical trial model, which might benefit both human and veterinarian patients [39]. Nevertheless, in spite of the high value of the canine mammary carcinoma model, extrapolation of results to humans still requires additional *in vitro* work in human cancer. In the present work we used cell lines isolated from spontaneously occurring canine mammary tumors and taking advantage of this *in vitro* model and its xenograft tumors, we evaluated the effects of oseltamivir phosphate on mammary tumor progression and invasion.

The viral sialidase inhibitor, oseltamivir phosphate, has often been reported not to affect the activity of mammalian endogenous sialidases (Neu1, Neu2, Neu3 and Neu4) [8, 20]. Nevertheless, our data in canine mammary tumor cells suggests the opposite. Oseltamivir phosphate inhibited endogenous sialidase activity in CMA07 and CMT-U27 cells (assessed by the amount of MuNana metabolized, a substrate used as a conventional method for the measurement of sialidase activity [41]). This inhibition appeared to be dependent on oseltamivir phosphate concentrations. Our findings are in agreement with what was concluded in a recent systematic review on oseltamivir phosphate benefits and disadvantages, which states that there are potential harms that should be better investigated and further suggests that encountered contradictions might be explained by direct effect on the host’s endogenous neuraminidase [42]. Previous experimental studies attempting to assess mammalian sialidase impairment in the presence of viral sialidase inhibitors were performed using mainly recombinant sialidases and normal immortalized epithelial cells [7, 8]. However, other studies [43] show that Neu1 and Neu4 sialidase activity is inhibited in the presence of oseltamivir phosphate *in vitro*. These somewhat contradictory results may be explained by the specificities of each mammalian sialidase, the microenvironment to which they are exposed and the cell type in which studies have been conducted. Recently, Tsai *et al*. have elegantly shown that mammalian sialidase inhibition is enhanced due to subtle protein structural differences and also due to differences in pH sensitivity [41]. Moreover, tumor cells present up-regulated expression of carboxylesterase 1 when compared with non-neoplastic cells [44]. Carboxylesterase 1 is necessary for the activation of oseltamivir phosphate. This cannot be excluded as one of the reasons for the surprising oseltamivir phosphate biological effects found in the present work, when compared with data from other groups carried out in non-tumor cells [45].

Oseltamivir phosphate treatment led to increased cell migration rate towards wound closing. This was statistically significant and also dose-dependent in the CMT-U27 cells. Furthermore, oseltamivir phosphate treatment was significantly associated with a higher number of CMT-U27 cells invading an extracellular matrix (ECM). The effect of oseltamivir phosphate on migration also seemed to be dose-dependent. Our results are therefore in line with studies showing a 30 to 40% cell invasion decrease upon overexpression of sialidases [46]. No significant differences were observed regarding the benign cell line CMA07 migration capacity. This is likely to be due to contributing migration mechanisms other than sialylation present in this cell line. Sialylation is a feature closely related to malignancy. As such this result comes as a further evidence to the importance of sialylation in the dissemination capacity of metastatic mammary cells.

Several reports showed the importance of sialylated structures for cancer cell migrating and invading capability *in vitro* [19, 24]. Therefore, we then hypothesized that the mechanism responsible for the observed increase in oseltamivir phosphate-induced mammary cell invasiveness could be an increase in the sialylation levels of most (or at least some) glycoconjugates. In order to assess this, we used plant lectin-based approaches. Oseltamivir phosphate increased α2,6 type glycan structures in CMA07 and CMT-U27 protein extracts. This was particularly evident when sorting proteins not only by molecular weight but also by isoelectric point. This may probably be related to sialic acid monosaccharides being negatively charged at physiologic pH. Furthermore, increased α2,6 type sialylation was even more expressive when looking at the fluorescent analysis of all cell glycoconjugates. Sialic acids may be added to glycolipids as well as to glycoproteins thereby likely justifying the later observation [47]. Unlike what happened regarding α2,6, there was no increase in α2,3 type structures. This might be explained by the previously reported ability of MAL I and MAL II to recognize unsialylated galactose terminal structures as well as α2,3 terminal sialic acid structures. This prompted us to analyze the expression of specific glycans involved in cancer progression. Normal breast tissue commonly expresses unsialylated structures such as Le(x), mainly in the apical part of cell ducts [48]. Sialyl-Lewis (SLe) antigens, such as SLe(x), SLe(a) are structures with α2,3-linked sialic acids found on glycolipids and glycoproteins of normal leucocytes and in tumor but rarely on non-tumor epithelial cells. High SLe antigens’ expression is significantly associated to metastatic breast cancer [49]. Thomsen nouvelle (Tn) antigen can be sialylated resulting in the synthesis of the dissaccharide Neu5Acα2,6GalNAc-R, STn, which is absent in normal healthy tissues but is detected in almost all kinds of carcinomas [19]. STn antigen expression has been associated with cancer aggressiveness and is currently being studied as a therapeutical target in breast cancer patients [50]. Analyzing SLe(x) and STn glycans in particular showed that SLe(x) expression was increased in a specific subset of proteins of CMA07 and CMT-U27 cells treated with oseltamivir phosphate. This reinforced the idea that oseltamivir phosphate does not induce a generalized increased in α2,6 sialylation.

Having observed oseltamivir phosphate effects in CMT-U27 cells *in vitro*, we aimed to evaluate its potential efficacy in facilitating invasion and whether or not this would be related to altered sialylation *in vivo*. In order to do so, mice were treated with oseltamivir phosphate several times higher than that which would be expected to inhibit only viral sialidases [42]. CMT-U27 is a highly aggressive and metastatic mammary cell line [32]. Oseltamivir phosphate-treated mice presented significantly more inflammation in their primary tumors. Changes in tumor cell sialylation may well affect the activity of siglec-expressing immune cells and thus influence the numbers of infiltrating inflammatory cells in a given tumor [51, 52]. At the end of the *in vivo* experiment, only one of the control animals presented well-established distant metastases while all four oseltamivir phosphate-treated nude mice presented histopathologically confirmed lung metastases. Although the *in vivo* experiment involved a limited numbered of animals in the study groups, it was enough to reach statistical significance for evaluation of inflammation and, most importantly, provide results indicative of a tendency toward increased metastases. Our results are in agreement with previous findings on a highly metastatic NL17 mouse melanoma cell line, which upon sialidase overexpression showed a marked suppression of lung metastasis [53]. Moreover, several studies pointed out a crucial contribution of altered sialylation to the metastatic tumor cell phenotype *in vivo* [17, 54]. In our work, SNA-binding showed that oseltamivir phosphate treatment increases α2,6 type glycan structures in CMT-U27 xenografts also in agreement with the *in vitro* findings. No clear differences were observed concerning α2,3 type glycans expression in CMT-U27 cells *in vitro* and *in vivo*. In tumor cells, unlike α2,3 linked sialic acids, α2,6 type glycan structures play a major biological role related to the galectins’ family. An important activity of the cell surface α2,6 type sialylation is to block galectin-induced tumor cell-cell and cell-ECM adhesion, particularly important for primary tumor cell detachment and intravascular homotypic aggregation, thereby facilitating tumor cell invasion [55]. We had previously shown that sialylation impairs galectin-3 binding to canine mammary tumor cells and hypothesized that this might be relevant in regulating adhesive/de-adhesive events in the acquisition of invasive capacity by metastatic mammary cells [34]. It is of note that despite differences in sialylation having been observed in the present work, when treating the animals with oseltamivir phosphate, its dose was established empirically. As such, correlations between *in vitro* and *in vivo* drug exposures are presently unknown.

Despite non-significant, a slight increase of SLe(x) expression in the tumors of oseltamivir phosphate-treated animals was also observed. Mounting evidence relates increased SLe(x) expression to metastatic cancer cell behavior. For exemple, SLe(x) expression levels are increased in tumors of patients presenting distant metastasis when compared with non-metastatic patients [56]. In addition, our group has recently shown that overexpressing a sialyltransferase, ST3GAL4, in gastric cancer cells leads to SLe(x) antigen expression which in turn induces a more invasive phenotype [24]. MUC1 mucin is a key player in canine and human mammary cancer metastasis [57, 58]. Increased MUC1 sialylation is thought to be responsible for decreased cell-cell adhesion and thus facilitate tumor cell detachment from primary sites. In addition, it binds endothelial selectins *via* SLe antigens, promoting hematogenous dissemination of cancer cells [58]. In fact, we have previously shown that canine mammary tumor cells invading the vasculature express MUC1 (weighting approximately 120 kDa) at focal adhesions, reinforcing its role in cell motility [34]. In addition, impairing SLe/selectin interactions has been demonstrated to decrease lung metastasis *in vivo* [59]. And finally, disaccharide decoy molecules to reduce overall sialylation proved to reduce lungs metastases in experimental studies *in vivo* [25].

Aberrant sialylation is considered one of the first hallmarks of malignancy. Overall, increased contents of sialic acids in glycoconjugates are associated with cancer progression, metastasis and poor patient survival [15]. Although, modulating cancer cells’ sialylation has been found to be difficult due to the complexity of glycan synthesis with an immense array of glycosyltransferases including (but not exclusively) sialyltransferases involved in the process [23, 60]. In the present work we provide evidence supporting the hypothesis that oseltamivir phosphate inhibits mammalian sialidase activity. As such, this study may open a new avenue of research regarding sialidases as main modulators of sialylation-related mechanisms of invasion in mammary cell tumors.

## Supporting Information

S1 Fig Cell confluence during oseltamivir phosphate treatment. CMA07 and CMT-U27 cell confluence in the beginning (T0) and 7 days upon treatment with different concentrations of oseltamivir phosphate, 0.305 μM 3.05 μM and 30.5 μM. PBS was used as control. Analysis of cell confluence and morphology were performed. Despite no major differences being observed regarding the morphology of CMT-U27 cells. CMA07 cells treated with oseltamivir phosphate presented a rounder shape at times, which is never observed in non-treated cells which are spindle shaped. Photographs were taken using a contrast inverted microscope (x20 magnification).
(TIF)

S2 Fig Siaα2,3Galβ1,4GlcNAc and Siaα2,3Galβ1,3GlcNAc terminal structures in CMA07 and CMT-U27 cell lines upon oseltamivir phosphate treatment. **(A)** Expression of terminal sialylated structures was evaluated by lectin blot analysis with MAL I and MAL II plant lectins. In MAL I lectin blot, no differences were observed in α2,3 sialic acid structures in proteins from both CMA07 and CMT-U27 cells treated with oseltamivir phosphate when compared with control (PBS). Regarding MAL II lectin blot there was an increase in terminal α2,3 sialic acid structures expression in proteins from lysates of CMT-U27 cells treated with 0.305 μM, 3.05 μM oseltamivir phosphate, in a molecular weight of about 120 kDa. **(B)** Terminal Siaα2,3Galβ1,4GlcNAc structures in CMA07 and CMT U27 cell lines was assessed using MAL I and MAL II plant lectins fluorescent labeling. CMA07 cells treated with oseltamivir phosphate (x20 magnification) do not present alteration in Siaα2,3Galβ1,4GlcNAc and Siaα2,3Galβ1,3GlcNAc expression. CMT-U27 cells treated with 3.05 μM oseltamivir phosphate showed slightly increased expression of Siaα2,3Galβ1,4GlcNAc and Siaα2,3Galβ1,3GlcNAc when compared to non-treated cells.
(TIF)

S3 Fig Relative tumor volume upon oseltamivir phosphate treatment. CMT-U27 cells were inoculated into the mammary gland fat pad of female nude mice (N:NIH(S)II-*nu/nu*). A control group (n = 4) and an oseltamivir phosphate treated group (n = 4) were used. Tumor growth was measured over time during oseltamivir phosphate treatment. During 40 consecutive days oseltamivir phosphate-treated mice were treated IP with 100 mg/Kg of oseltamivir phosphate. Despite the fact that no significant differences in tumor growth were observed between the two groups of mice, there was a tendency towards a higher relative tumor volume in oseltamivir phosphate-treated mice.
(TIF)

S4 Fig Peritoneal Invasion in oseltamvir phosphate treated mice. Pictures show carcinomatous effusion and peritoneal cavity invasion present in two oseltamivir phosphate treated mice.
(TIF)
