# Supplementary figures and images for: Anti-Influenza Neuraminidase Inhibitor Oseltamivir Phosphate Induces Canine Mammary Cancer Cell Aggressiveness
Source: PLoS One. 2015 Apr 7;10(4):e0121590. doi: 10.1371/journal.pone.0121590 (PMC4388625; doi:10.1371/journal.pone.0121590)

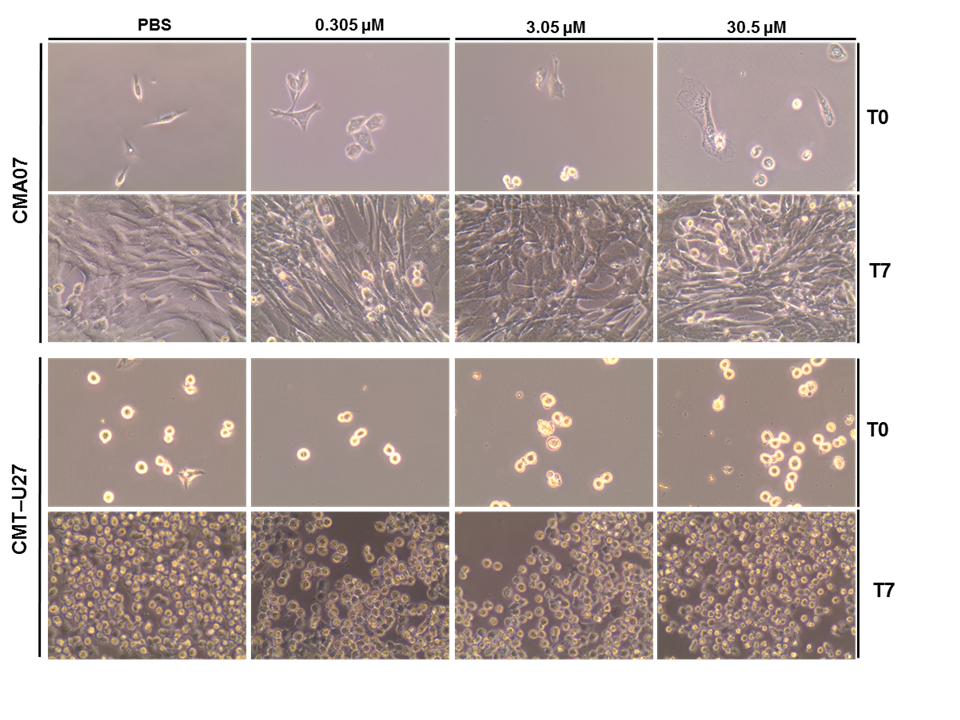

Supplement: S1 Fig — CMA07 and CMT-U27 cell confluence in the beginning (T0) and 7 days upon treatment with different concentrations of oseltamivir phosphate, 0.305 μM 3.05 μM and 30.5 μM. PBS was used as control. Analysis of cell confluence and morphology were performed. Despite no major differences being observed regarding the morphology of CMT-U27 cells. CMA07 cells treated with oseltamivir phosphate presented a rounder shape at times, which is never observed in non-treated cells which are spindle shaped. Photographs were taken using a contrast inverted microscope (x20 magnification). (TIF) [file pone.0121590.s001.tif]

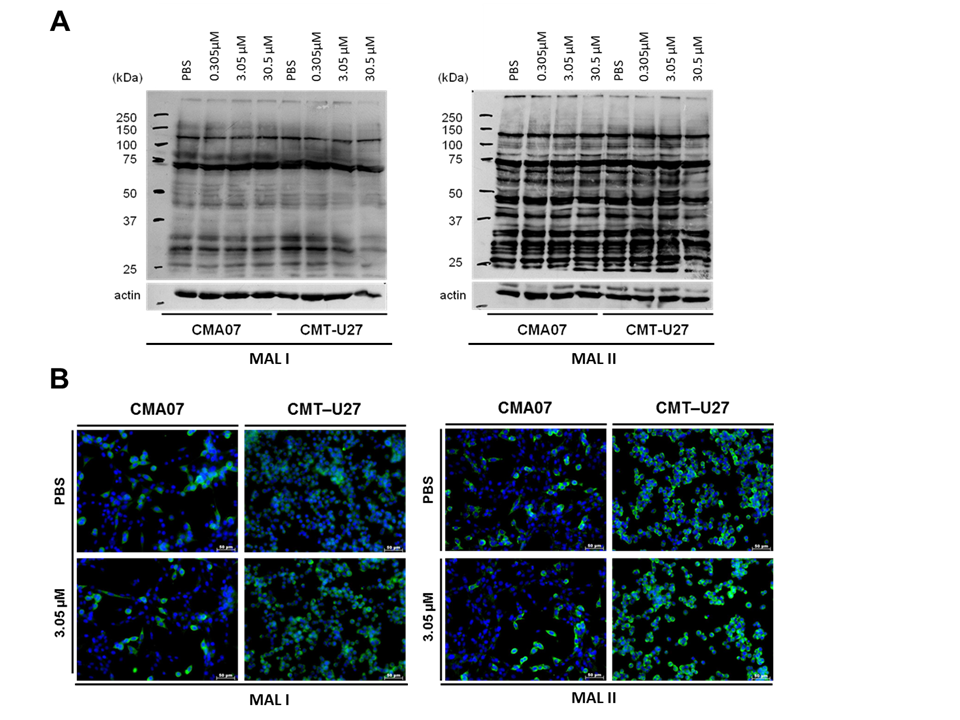

Supplement: S2 Fig — (A) Expression of terminal sialylated structures was evaluated by lectin blot analysis with MAL I and MAL II plant lectins. In MAL I lectin blot, no differences were observed in α2,3 sialic acid structures in proteins from both CMA07 and CMT-U27 cells treated with oseltamivir phosphate when compared with control (PBS). Regarding MAL II lectin blot there was an increase in terminal α2,3 sialic acid structures expression in proteins from lysates of CMT-U27 cells treated with 0.305 μM, 3.05 μM oseltamivir phosphate, in a molecular weight of about 120 kDa. (B) Terminal Siaα2,3Galβ1,4GlcNAc structures in CMA07 and CMT U27 cell lines was assessed using MAL I and MAL II plant lectins fluorescent labeling. CMA07 cells treated with oseltamivir phosphate (x20 magnification) do not present alteration in Siaα2,3Galβ1,4GlcNAc and Siaα2,3Galβ1,3GlcNAc expression. CMT-U27 cells treated with 3.05 μM oseltamivir phosphate showed slightly increased expression of Siaα2,3Galβ1,4GlcNAc and Siaα2,3Galβ1,3GlcNAc when compared to non-treated cells. (TIF) [file pone.0121590.s002.tif]

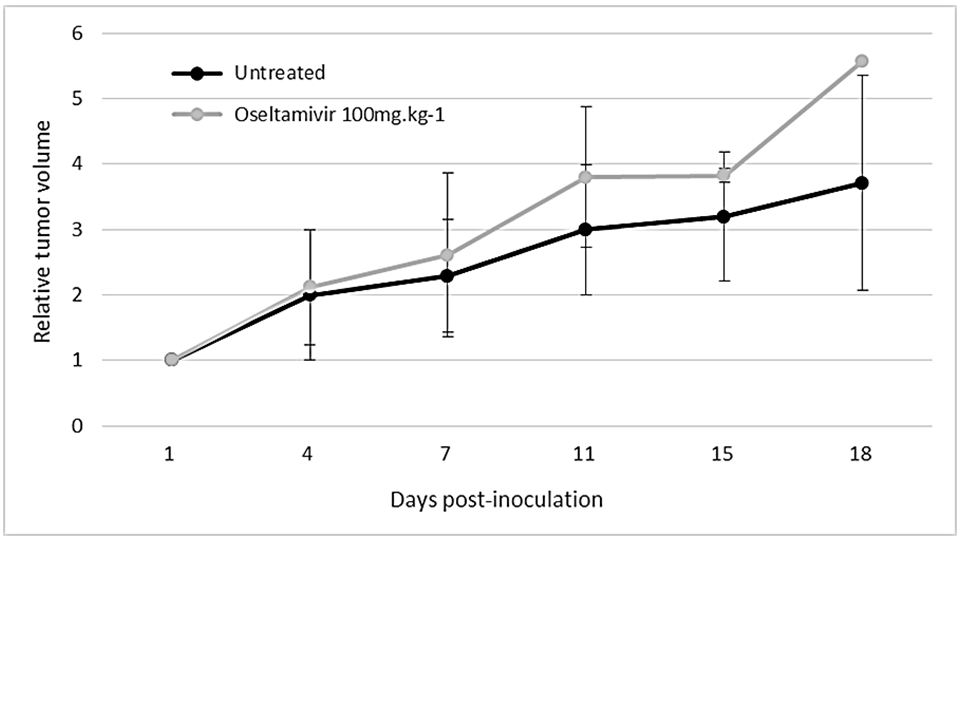

Supplement: S3 Fig — CMT-U27 cells were inoculated into the mammary gland fat pad of female nude mice (N:NIH(S)II-nu/nu). A control group (n = 4) and an oseltamivir phosphate treated group (n = 4) were used. Tumor growth was measured over time during oseltamivir phosphate treatment. During 40 consecutive days oseltamivir phosphate-treated mice were treated IP with 100 mg/Kg of oseltamivir phosphate. Despite the fact that no significant differences in tumor growth were observed between the two groups of mice, there was a tendency towards a higher relative tumor volume in oseltamivir phosphate-treated mice. (TIF) [file pone.0121590.s003.tif]

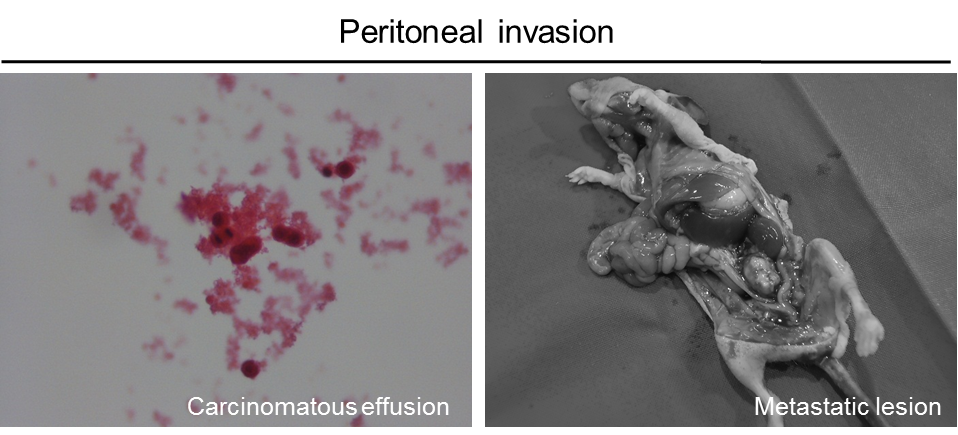

Supplement: S4 Fig — Pictures show carcinomatous effusion and peritoneal cavity invasion present in two oseltamivir phosphate treated mice. (TIF) [file pone.0121590.s004.tif]
